# SynthEco - A multi-layered digital ecosystem for analysing complex human behaviour in context

**DOI:** 10.23889/ijpds.v8i3.2285

**Published:** 2023-09-11

**Authors:** Antonia Gieschen, Catherine Paquet, Raja Sengupta, Anna-Liisa Aunio, Fares Belkhiria, Shawn Brown, Laurette Dube

**Affiliations:** 1 University of Edinburgh; 2 Université Laval; 3 McGill University; 4 Dawson College

## Introduction & Background

Human behaviour is multi-faceted and complex, with different dimensions interacting and impacting each other and individuals operating in an environmental context. In order to understand this behaviour better, the combination of data from different sources is useful to uncover some of those interactions and complexities. We present a multi-layered digital ecosystem based on a data platform providing statistically representative synthetic population derived from census data at different geo-spatial granularity, which we call SynthEco. This platform is enriched with individual data stemming from cohorts and cross-sectional surveys and geo-scanning of different layers of socio-environmental actors and conditions to create a complex digital ecosystem.

## Objectives & Approach

The objective of SynthEco is to allow for the analysis of behaviour, as well as health and wellbeing outcomes, through the integration of cohort and cross-sectional data into a geospatially anchored synthetic population embedded into environmental data which is forming the backdrop. We demonstrate the use of this platform on the example of Montreal, Canada. The synthetic population is first generated from census data using iterative proportional fitting, which allows for the creation of a population data set that is artificial yet statistically representative for a given geospatial granularity, such as a city. Each individual household is assigned a geospatial location, which allows for the consideration of their surrounding environment including enterprises or institutions such as schools, hospitals and the local food environment. Through fuzzy matching and statistical extrapolation, different cohort and cross-sectional survey data are then merged to individual records, in order to describe them in more detail. This includes health, as well as financial wellbeing or social environment descriptors.

## Relevance to Digital Footprints

There are two important points made through the presented work in relation to Digital Footprints data: the first is the technical approach to merging multiple datasets describing different dimensions of interacting human characteristics and behaviour by anchoring them into a synthetic population through fuzzy record matching. The second is the consideration of a spatial dimension when describing human behaviour. This is especially important when describing behaviour within local environments, such as the interaction with local food outlets.

## Results

Recent work in this context includes an analysis of the food environment in Montreal, Canada. It introduces a way of utilising the synthetic population to predict the healthfulness of their local environment in terms of healthy food outlets, as well as providing a platform for the analysis of food environment surveillance and intervention simulations. For this purpose, the healthfulness of different census tract regions in Montreal is calculated to identify food deserts, food swamps, as well as healthy areas as defined through the Modified Retail Food Environment Index. We test different machine learning approaches to then predict these healthfulness scores using census variables from the synthetic population in their respective census tract, achieving accuracy scores of around 0.53 to 0.60. This demonstrates that census data has some limited predictive power in explaining the healthiness of the local food environment, which could be especially relevant for situations in which no information on the retailers is available to local policy makers. Future work can extend this approach to also include further data describing the population, stemming from the integrated cohorts and survey data, which could improve the prediction accuracy or help in identifying areas of concern.

## Conclusions & Implications

The presented SynthEco platform views individuals as agents nested within modular systems of systems, trying to capture both internal systems and processes as well as environmental ones within which individuals are operating. The platform thus enables the application of computational systems modelling for the analysis of individual human behaviour in contexts. As demonstrated through the example of using SynthEco in the context of healthier food environments, the approach is especially relevant to practitioners and policy makers interested in local intervention strategies and identifying areas for targeted policy related to different dimensions of health and wellbeing.

